# Deep Gray Matter Iron Content in Neuromyelitis Optica and Multiple Sclerosis

**DOI:** 10.1155/2020/6492786

**Published:** 2020-05-19

**Authors:** Adam Pudlac, Andrea Burgetova, Petr Dusek, Petra Nytrova, Manuela Vaneckova, Dana Horakova, Jan Krasensky, Lukas Lambert

**Affiliations:** ^1^Department of Radiology, First Faculty of Medicine, Charles University and General University Hospital in Prague, Katerinska 32, Prague 128 08, Czech Republic; ^2^Department of Neurology and Centre of Clinical Neuroscience, First Faculty of Medicine, Charles University and General University Hospital in Prague, Katerinska 32, Prague 128 08, Czech Republic

## Abstract

**Background:**

Neuromyelitis optica (NMO) and multiple sclerosis (MS) are often presenting with overlapping symptoms. The aim of this study was to determine whether and how NMO and MS differ regarding cerebral iron deposits in deep gray matter (DGM) and the correlation between iron deposition and clinical severity as well as to regional atrophy of the DGM.

**Methods:**

We analyzed 20 patients with NMO, 40 patients with a relapsing-remitting (RR) form of MS, and 20 healthy controls with 1.5T MRI. Quantitative susceptibility mapping (QSM) was performed to estimate iron concentration in the DGM.

**Results:**

Patients with NMO have higher magnetic susceptibility values in the substantia nigra compared to healthy controls. RRMS patients have lower magnetic susceptibility values in the thalamus compared to healthy controls and NMO patients. Atrophy of the thalamus, pulvinar, and putamen is significant both in RRMS compared to NMO patients and healthy controls. A correlation was found between the disability score (EDSS) and magnetic susceptibility in the putamen in RRMS.

**Conclusions:**

This study confirms that a disturbed cerebral iron homeostasis in patients with NMO occurs in different structures than in patients with RRMS. Increased magnetic susceptibility in substantia nigra in NMO and decreased magnetic susceptibility within the thalamus in RRMS were the only significant differences in the study sample. We could confirm that iron concentration in the thalami is decreased in RRMS compared to that in the HC group. Positive association was found between putaminal iron and EDSS in RRMS.

## 1. Introduction

MS and neuromyelitis optica (NMO) are inflammatory diseases of the central nervous system presenting with overlapping symptoms; therefore, their differential diagnosis cannot be based on clinical examination alone. The presence of aquaporin-4 immunoglobulin G (AQP4-IgG) in sera of NMO patients definitely distinguishes these two diseases [1]. Aquaporin-4 (AQP4) as a target antigen is expressed on the cell membrane of astrocytes and ependymal cell. Binding of AQP4-IgG to AQP4 can cause complement activation with subsequent cell destruction or internalization of AQP4 resulting in an impairment of osmotic balance. The majority of NMO patients have brain lesions, especially in areas with a high expression of AQP4 (diencephalic lesions surrounding the third ventricles and cerebral aqueduct, dorsal brainstem lesions adjacent to the fourth ventricle, etc.); however, their morphology and location differ from lesions typically found in MS [2, 3]. Both entities also differ in pathology, in autoimmune mechanisms, and in the response to some immunotherapies. Neuroinflammatory and neurodegenerative changes in multiple sclerosis (MS) are associated with changes in cerebral iron accumulation as documented in MRI and neuropathological studies [4, 5]. Abnormal iron deposits have been detected in the deep gray matter (DGM), i.e., in the putamen, caudate nucleus, and globus pallidus and in a subset of white matter (WM) lesions using iron-sensitive MR techniques such as R2∗ relaxometry, magnetic field correlation imaging, phase imaging, and quantitative susceptibility mapping (QSM) [6–8]. On the contrary, WM lesions in NMO patients do not contain increased iron deposits [9]. Previous studies brought inconsistent results as to whether there are any differences in the iron content of DGM structures between MS and NMO [4, 10].

The primary goal of this study was to compare iron content in DGM among patients with MS, NMO, and healthy subjects using QSM. The secondary goals were to investigate the correlation between iron deposition and clinical severity and to assess regional atrophy of the DGM using volumetric analysis in both patient groups.

## 2. Materials and Methods

This cross-sectional study was performed in accordance with the Declaration of Helsinki, it was approved by the Ethics Committee of the General Faculty Hospital and First Medical Faculty, Charles University, and all participants signed informed consent. Between December 2013 and March 2015, 20 patients with NMO underwent MRI of the brain.

### 2.1. Study Subjects

An age- and sex-matched cohort of 20 healthy controls (1 : 1) and 40 patients with a relapsing-remitting (RR) form of MS (1 : 2) was examined within the same time frame. The group of RR MS and controls is partially overlapping with subjects included in our previous study [7]. All patients with RRMS fulfilled the revised McDonald criteria [11], and the diagnosis of NMO was based on Wingerchuk's diagnostic criteria [12]. Patients were examined by a specialist in demyelinating disorders. Neurological disability was evaluated by Kurtzke disability status scale (EDSS). All 20 NMO patients were AQP4-IgG positive; all samples were tested by commercially available immunofluorescence cell-based assay (CBA) using recombinant human M1-AQP4 (Euroimmun, Lübeck, Germany). Demographic data of the patients are shown in Table 1.

### 2.2. MRI

The examinations were performed using a 1.5T MR imaging system (Gyroscan NT; Philips Healthcare, Best, the Netherlands); a standard quadrature head coil was used. The protocol included FLAIR (150 axial sections, TR 1000 ms, TE 140 ms, TI 2600 ms, spatial resolution 1 × 1 × 1 mm^3^, scan duration 10 minutes and 16 seconds), T1-weighted imaging (fast-field echo/3D, 150 axial sections, TR 25 ms, TE 5.01 ms, spatial resolution 1 × 1 × 1 mm^3^, scan duration 12 minutes and 48 seconds), and susceptibility-weighted imaging (fast-field echo/3D, 100 axial sections, TR 48.1 ms, TE 33.2 ms, spatial resolution 0.8 × 0.8 × 2.0 mm^3^, scan duration 6 minutes and 30 seconds) pulse sequences.

### 2.3. Image Analysis

**Figure 1:**
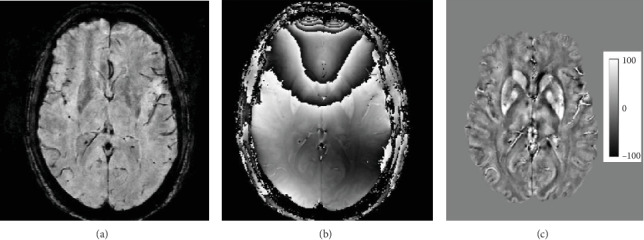
Example axial MR image slice depicting gradient-echo magnitude (a), raw phase (b), and quantitative susceptibility map (c). Magnetic susceptibility scale in ppb is shown.

**Figure 2:**
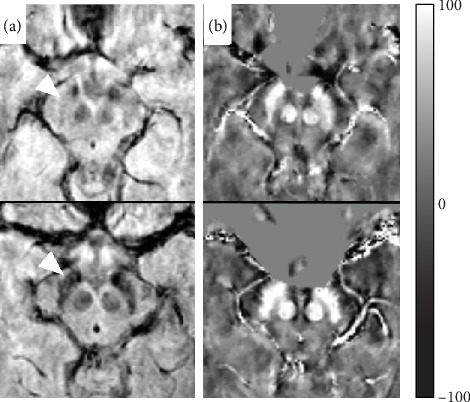
Comparison of axial images at the level of mesencephalon; gradient-echo magnitude (a) and quantitative susceptibility map (b) of a typical healthy control (upper row) and typical age-matched neuromyelitis optica patient (bottom row). The substantia nigra is denoted by white arrowhead. Magnetic susceptibility scale in ppb is shown.

QSM images were reconstructed using a total generalized variation method as previously described [13]. Briefly, the reconstruction consisted of Laplacian unwrapping, background field removal, and dipole inversion by using the total generalized variation regularization in a single integrated step. QSM images were rigidly aligned with the T1-weighted images [7, 14]. Measurements of the volume and regional median bulk susceptibilities in parts per billion (ppb) were performed in the following regions of interest (ROIs) segmented on the T1-weighted images using the automated algorithm included in FreeSurfer, version 4.5 (http://surfer.nmr.mgh.harvard.edu/): globus pallidus (GP), putamen (Put), caudate nucleus (CN), and thalamus (Thal) (Figure 1). Additionally, the volume of the following structures was manually segmented on anonymized QSM images by a senior radiologist blinded to the diagnosis: pulvinar thalami (PT), nucleus ruber (NR), and the substantia nigra (SN); median bulk susceptibilities were extracted (Figure 2). QSM values were adjusted to a manually drawn ROI in the occipital white matter avoiding any lesions. The volume of cerebral structures was normalized using the residual approach method, which uses linear regression between the volume of the structure and the brain envelope [15].

### 2.4. Statistical Analysis

Statistical analysis was performed using SPSS 19 (IBM Corp., Armonk, NY) and R (the R Foundation for Statistical Computing, Vienna, Austria). Matching of the groups was performed using the MatchIt function in R. To test for statistical significance among the study groups, we used ANOVA with Tukey's HSD post hoc tests, the Kruskal-Wallis test with Dunn's post hoc tests, and *χ*^2^ test as appropriate. Correlation between EDSS and QSM was expressed as Spearman's rho coefficient. Two-tailed *p* values below 0.05 were considered significant.

## 3. Results and Discussion

### 3.1. Comparison of Magnetic Susceptibility among NMO, RRMS, and HC

NMO had higher bulk magnetic susceptibility values in the SN (107.2 ± 19.6) compared to healthy controls (91.0 ± 16.1, *p* = 0.030) but not to RRMS (95.3 ± 22, *p* = 0.081). RRMS had lower susceptibility in the thalamus (10.8 ± 5.6) compared to NMO (16.3 ± 6.3, *p* = 0.0086) and HC (16.1 ± 8.2, *p* = 0.011) (Table 2).

### 3.2. Volume of DGM, Comparison of NMO, RRMS, and HC

The volume of the thalamus was smaller in RRMS patients (13.4 ± 1.2 ml) compared to HC (14.3 ± 0.7 ml, *p* = 0.0094) and NMO (14.2 ± 1.2 ml, *p* = 0.023). Significantly decreased volume in RRMS patients was also noted in the putamen and pulvinar, compared to NMO and RR (Table 3).

### 3.3. Association between Magnetic Susceptibility and Age

There was a significant correlation of magnetic susceptibility values with age in the nucleus ruber (*r* = 0.45, *p* = 0.0040), the caudate nucleus (*r* = 0.49, *p* = 0.0013), and the putamen (*r* = 0.51, *p* = 0.0007) in RRMS. In NMO, the correlation of magnetic susceptibility with age was not significant in any of the evaluated structures.

### 3.4. Association between Magnetic Susceptibility and Disability

A weak correlation was found between the EDSS disability score and magnetic susceptibility in the putamen (*r* = 0.32, *p* = 0.046) in RRMS only (Table 4).

This study showed that patients with NMO have higher magnetic susceptibility values in the substantia nigra compared to healthy subjects. RRMS patients have lower magnetic susceptibility values in the thalamus and greater atrophy of the putamen, thalamus, and pulvinar compared to healthy subjects and NMO patients. A positive correlation was found between the disability score (EDSS) and magnetic susceptibility in the putamen in RRMS.

In this work, we have confirmed that the pattern of iron deposition is different in NMO compared to RRMS patients. Notably, NMO patients have higher magnetic susceptibility in SN whereas other basal ganglia in NMO patients have susceptibility values comparable to healthy subjects. On the contrary to our result, Chen et al. [4] observed increased iron deposition in the bilateral SN in MS patients compared to the NMO and control groups. There are several possible reasons for this discrepancy. Firstly, compared to previous studies, NMO patients in our study are considerably older with longer disease duration. In the theoretical case of a steeper slope of age-related iron accumulation in NMO, the difference between patients and healthy controls may be apparent only in older subjects. Secondly, QSM may be superior in detecting abnormal iron deposits compared to other MR methods, such as phase imaging or R2∗ relaxometry, used in some previous studies [16]. Lastly, there is a difference between SN pars reticulata and pars compacta which have distinct iron levels and its concentration is regulated differently in these subregions [17]. Our segmentation based on QSM images also included the caudal layers of SN and could thus theoretically contain more of SN pars compacta. NMO is a relatively rare disorder, and therefore, previous studies targeting iron homeostasis in NMO included low number of patients. Doring et al. found in a retrospective study of 6 female and 6 male patients with NMO that susceptibility values were decreased in the nucleus ruber with a greater difference in older subjects [10]. In our study, which included higher number of NMO patients with longer disease duration and which was better balanced in terms of the gender prevalence of MS, we were only able to account for age-dependent changes but could not confirm any between-group differences in magnetic susceptibility of the nucleus ruber. Unfortunately, Doring et al. did not include SN in their analysis. Nevertheless, previous studies using diffusion tensor imaging (DTI) also suggested gray matter abnormalities in NMO [18] and abnormal pattern of movement-associated cortical activations in NMO depicted by a functional MRI study supported gray matter involvement in the disease [19].

Iron metabolism might reflect the chronic oxidative injury in NMO patients. It is still unclear whether iron metabolism is implicated in the pathogenesis of NMO [19]. The finding of isolated iron accumulation in SN resembles the pattern observed in Parkinson's disease (PD). Interestingly, recent animal study showed high perivascular density of AQP4 in the SN that was further increased in the 1-methyl-4-phenyl-1,2,3,6-tetrahydropyridine (MPTP) model of PD [20].

In this study, we confirmed that iron concentration in the thalami is decreased in RRMS compared to the HC group. This is consistent with the results of recently published works [7, 8, 21].

Apart from the brain disease and its variants, course, and duration [21], numerous other factors that result in iron deposition abnormalities have been identified including gene polymorphisms associated with iron regulation [22, 23]. Moreover, mere magnetic susceptibility of the tissue does not perfectly account for its iron content because iron occurs in forms with different magnetic properties. However, correlation between tissue magnetic susceptibility and iron concentration has been established in ex vivo studies comparing MR, histopathologic sections, and measurement of iron concentration [16].

The magnetic susceptibility is not just a marker of the disease, but it is also independently associated with the clinical disability in MS patients. We showed that increased susceptibility in the putamen in RRMS patients is associated with higher EDSS. The correlation between iron concentration in putamen and clinical severity in MS is consistent with the results in previously published studies [24–26]. In contrast, Zivadinov et al. found a correlation between iron accumulation and disability only in globus pallidus [21]. Furthermore, he reported an association between disability and decreased iron in the thalamus.

This study showed that patients with MS have smaller volumes of the putamen, thalamus, and pulvinar compared to NMO and HC. Several studies have identified reduction in DGM volume in NMO patients [27–29]. The studies comparing regional GM atrophy in NMO and MS are rare and have inconsistent results. Although some studies found DGM atrophy in NMO restricted to the thalamus, the difference in our study was not significant [30, 31]. Nevertheless, patients with cognitive impairment exhibit a more severe atrophy. Decreased volume of the thalamus in NMO was shown in the study of Fan et al.; volume was smaller in the AQP4 (−) group and the MS group than that in the HC group [32]. Duan et al. found atrophy in NMO patients in several regions of frontal, temporal, parietal lobes, and insula but not in the DGM [27].

This study has several limitations. Firstly, the number of NMO patients enrolled in the study is relatively small given the paucity of this autoimmune disease. Secondly, we did not evaluate susceptibility changes in inflammatory lesions as has been previously done by other research groups using a 7T MR [2]. Thirdly, cross-sectional design provides no information regarding the temporal dynamics of iron accumulation. Iron deposition within white matter and inflammatory lesions should be the focus of further investigations as well as longitudinal settings of the study.

## 4. Conclusions

Our study confirms that a disturbed cerebral iron homeostasis in patients with NMO occurs in different structures than in patients with RRMS. Increased magnetic susceptibility in substantia nigra in NMO and decreased magnetic susceptibility within the thalamus in RRMS were the only significant differences in the study sample. We could confirm that iron concentration in the thalami is decreased in RRMS compared to that in the HC group. Positive association was found between putaminal iron and EDSS in RRMS. The iron metabolism might reflect the chronic oxidative injury. It is still unclear whether iron metabolism is implicated in the pathogenesis of NMO. The magnetic susceptibility is not just a marker of the disease, but it is also independently associated with the clinical disability; positive association was found between putaminal iron and EDSS in RRMS. However, as thalamic iron concentration shows a specific pattern of evolution in time, only longitudinal studies may support this association.

## Figures and Tables

**Table 1:** Demographic and clinical data in NMO, RRMS, and HC groups.

	NMO	RRMS	HC	p
No. of subjects	20	40	20	—
Gender (male)	4 (20%)	8 (20%)	4 (20%)	1.0
Age (years)	48 ± 10	47 ± 9	50 ± 8	0.54
Disease duration (years)	11.4 ± 8.7	14.1 ± 8.2	—	0.25
EDSS (points)	4.5 ± 1.2^a^	3.1 ± 1.6^a^	0.0 ± 0.0^a^	<0.0001
Annual relapse rate	0.5 (0-3)^b^	0.0 (0-2)^b^		0.058

^a^All between-group difference significant (pairwise comparison), ^b^median (range). Abbreviations: NMO: neuromyelitis optica; RRMS: remittent-relapsing multiple sclerosis; HC: healthy controls; EDSS: expanded disability status scale. Unless otherwise indicated, data are presented as mean ± standard deviation.

**Table 2:** Mean adjusted magnetic susceptibility (in ppb) in deep gray matter structures.

	Mean susceptibility (±SD) (ppb)				Post hoc tests		
	HC	NMO	RRMS	*p*	HC-NMO	HC-RRMS	NMO-RRMS
Globus pallidus	83.7 ± 11.1	91.0 ± 19.2	83.6 ± 16.1	0.21			
Nucleus ruber	67.4 ± 17.4	71.7 ± 17.8	66.6 ± 19.5	0.60			
Caudate nucleus	41.1 ± 10.1	36.9 ± 11.2	39.3 ± 10.8	0.59			
Pulvinar thalami	35.0 ± 13.4	37.6 ± 11.2	32.9 ± 14.5	0.44			
Putamen	30.7 ± 7.4	26.5 ± 10.1	30.3 ± 11.7	0.34			
Substantia nigra	91.0 ± 16.1	107.2 ± 19.6	95.3 ± 21.6	0.030	0.032	0.69	0.081
Thalamus	16.1 ± 8.2	16.3 ± 6.3	10.8 ± 5.6	0.0019	0.90	0.011	0.0086

Abbreviations: NMO: neuromyelitis optica; RRMS: remittent-relapsing multiple sclerosis; HC: healthy controls.

**Table 3:** Adjusted volume (ml) of the deep gray matter structures.

	Mean volume (±SD) (ml)				Post hoc tests		
	HC	NMO	RR	*p*	HC-NMO	HC-RRMS	NMO-RRMS
Globus pallidus	3.40 ± 0.24	3.28 ± 0.39	3.13 ± 0.50	0.062			
Nucleus ruber	0.45 ± 0.11	0.45 ± 0.13	0.39 ± 0.11	0.122			
Caudate nucleus	6.91 ± 0.59	6.87 ± 0.60	6.81 ± 0.81	0.864			
Pulvinar thalami	1.46 ± 0.31	1.54 ± 0.35	1.07 ± 0.61	0.0008	0.847	0.0136	0.0023
Putamen	9.94 ± 0.75	10.02 ± 0.75	9.20 ± 0.91	0.0026	0.900	0.0194	0.0079
Substantia nigra	0.73 ± 0.15	0.76 ± 0.18	0.67 ± 0.16	0.124			
Thalamus	14.32 ± 0.73	14.22 ± 1.21	13.38 ± 1.24	0.0032	0.900	0.0094	0.023

Abbreviations: NMO: neuromyelitis optica; RRMS: remittent-relapsing multiple sclerosis; HC: healthy controls.

**Table 4:** Associations of EDSS with adjusted magnetic susceptibility values.

	NMO			RRMS	
	*r*_s_	*p*		*r*_s_	*p*
Globus pallidus	0.077	0.747		0.033	0.839
Nucleus ruber	0.109	0.647		0.245	0.128
Caudate nucleus	0.095	0.692		0.215	0.182
Pulvinar thalami	0.062	0.794		-0.090	0.580
Putamen	0.142	0.550		0.317	0.046
Substantia nigra	0.010	0.967		0.059	0.718
Thalamus	-0.064	0.789		0.120	0.462

*r*_s_ = Spearman's correlation coefficient; *p* = *p* value. Abbreviations: NMO: neuromyelitis optica; RRMS: remittent-relapsing multiple sclerosis.

## Data Availability

“The data used to support the findings of this study are available from the corresponding author upon request.”
